# Hybrid insulin peptide isomers spontaneously form in pancreatic beta-cells from an aspartic anhydride intermediate

**DOI:** 10.1016/j.jbc.2023.105264

**Published:** 2023-09-19

**Authors:** Samantha A. Crawford, Jason Groegler, Mylinh Dang, Cole Michel, Roger L. Powell, Anita C. Hohenstein, Kaitlin Reyes, Kathryn Haskins, Timothy A. Wiles, Thomas Delong

**Affiliations:** 1 Department of Pharmaceutical Sciences, Skaggs School of Pharmacy and Pharmaceutical Sciences, University of Colorado Anschutz Medical Campus, Aurora, Colorado, USA; 2 Department of Immunology and Microbiology, School of Medicine, University of Colorado Anschutz Medical Campus, Aurora, Colorado, USA

**Keywords:** aspartate (aspartic acid), beta cell (β-cell), type 1 diabetes, autoimmune disease, mass spectrometry, chromatography, insulin C-peptide, islet amyloid polypeptide, chromogranin A

## Abstract

Hybrid insulin peptides (HIPs) form in beta-cells when insulin fragments link to other peptides through a peptide bond. HIPs contain nongenomic amino acid sequences and have been identified as targets for autoreactive T cells in type 1 diabetes. A subgroup of HIPs, in which N-terminal amine groups of various peptides are linked to aspartic acid residues of insulin C-peptide, was detected through mass spectrometry in pancreatic islets. Here, we investigate a novel mechanism that leads to the formation of these HIPs in human and murine islets. Our research herein shows that these HIPs form spontaneously in beta-cells through a mechanism involving an aspartic anhydride intermediate. This mechanism leads to the formation of a regular HIP containing a standard peptide bond as well as a HIP-isomer containing an isopeptide bond by linkage to the carboxylic acid side chain of the aspartic acid residue. We used mass spectrometric analyses to confirm the presence of both HIP isomers in islets, thereby validating the occurrence of this novel reaction mechanism in beta-cells. The spontaneous formation of new peptide bonds within cells may lead to the development of neoepitopes that contribute to the pathogenesis of type 1 diabetes as well as other autoimmune diseases.

In type 1 diabetes (T1D), autoreactive CD4 T cells mediate the destruction of insulin-producing beta-cells in pancreatic islets. A long-standing question in T1D research is how the immune system gets tricked into attacking the body's own beta-cells. Post-translational modifications (PTMs) may provide an explanation for this loss of self-tolerance. PTMs that do not occur in the thymus may allow autoreactive T cells to evade the negative selection process and escape into the periphery, where they can reach beta-cells. Hybrid insulin peptides (HIPs) are PTM-induced epitopes that form through peptide bond formation between proinsulin fragments and other beta-cell peptides. HIPs contain nongenomic amino acid sequences not expressed in the thymus, making them plausible targets for pathogenic T cells in T1D. Various HIPs have been detected in human and murine islets through mass spectrometric analyses (1, 2, 3, 4, 5). For example, one subgroup of HIPs is generated through peptide bond formation between the carboxylic acid group of a specific leucine residue within insulin C-peptide and the N termini of various naturally occurring peptide cleavage products of proinsulin, chromogranin A (ChgA), or islet amyloid polypeptide (IAPP). In nonobese diabetic (NOD) mice, the primary animal model used for the study of T1D, CD4 T cells that trigger diabetes have been shown to target members of this subgroup of HIPs (1, 2, 6). We established that these HIPs form through proteolytic transpeptidation reactions mediated by the aspartic protease cathepsin D (7). HIP-reactive CD4 T-cell specificities have also been found in residual pancreatic islets of T1D organ donors, and significantly elevated levels of HIP-reactive CD4 T cells have been detected in peripheral blood mononuclear cells of T1D patients but not in nondiabetic controls (1, 8, 9). Another subgroup of HIPs, formed at an aspartic acid (D) residue within insulin C-peptide, has been detected in NOD islets. These HIPs contain the amino acid sequence EVED, derived from C-peptide, at their N-terminal side, linked to naturally occurring cleavage products of ChgA or IAPP (5, 10). Here, we show that the formation of these HIPs occurs spontaneously in beta-cells through an aspartic anhydride intermediate, yielding two HIP isomers: one containing a standard peptide bond and the other an isopeptide bond.

## Results

### Discovery of isoHIPs

**Figure 1:**
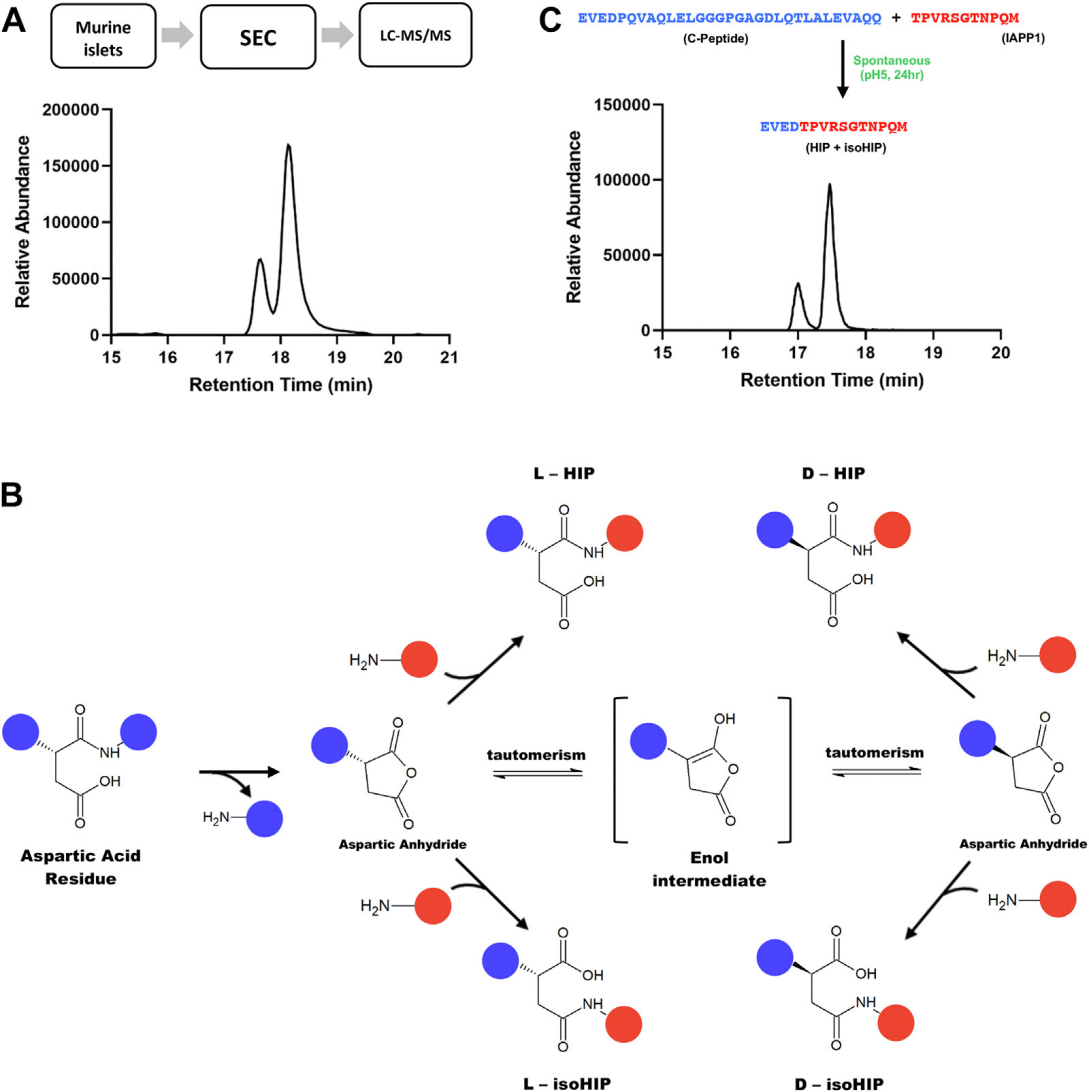
**Discovery of (iso)HIPs in NOD islets.***A*, extracted ion chromatogram (EIC) identifies two peaks representing the *m/z* of 830.38 for the peptide EVED-TPVRSGTNPQM present in the NOD islets. Schematic shows how NOD murine islets were subjected to fractionation by size-exclusion chromatography (SEC) and peptide-containing fractions were analyzed by mass spectrometry. Experiment was done in triplicate. Aforementioned data show one representative experiment. *B*, schematic showing the proposed mechanism for spontaneous (iso)HIP formation *via* an aspartic acid (D) residue. Four products of HIP formation are possible through tautomerization of an enol intermediate (L-HIP, D-HIP, L-isoHIP, and D-isoHIP). *C*, spontaneous *in vitro* formation of EVED-TPVRSGTNPQM. Full-length murine Ins2 C-peptide and a fragment of IAPP (IAPP1) were coincubated in the absence of protease/enzyme. EIC shows the results of the experiment identifying two peaks present in the reaction sample representing the *m/z* (830.38) for the peptide EVED-TPVRSGTNPQM. Experiment was done in triplicate. Aforementioned data show one representative experiment. HIP, hybrid insulin peptide; NOD, nonobese diabetic.

Using mass spectrometry (MS) on NOD islet extracts that were fractionated by size-exclusion chromatography (SEC), we identified multiple fragmentation spectra that were confidently matched to sequences in our HIP-database (Spectrum Mill Score >10.0; scored peak intensity >70%). Several of these HIPs form at the two D-residues that are present in murine C-peptide (Table 1). One of these HIPs contains the amino acid sequence EVED-TPVRSGTNPQM (HIP-junction is hyphenated), which forms between a fragment of C-peptide on the N-terminal side (EVED, *left peptide*) linked to a naturally occurring cleavage product of IAPP on the C-terminal side (TPVRSGTNPQM, *right peptide*). We had previously used MS to validate the presence of this HIP in NOD islets (5), a finding that was substantiated by others (10). Upon extracting an ion chromatogram (extracted ion chromatogram [EIC], *m/z* = 830.38) for this HIP, we noticed the presence of two closely adjacent peaks indicating the presence of two HIP-isomers (Fig. 1*A*). Because the new peptide bond of this HIP formed at a D-residue of the left peptide, we hypothesized that two HIP-isomers formed through a mechanism involving an aspartic anhydride intermediate (Fig. 1*B*). This intermediate is created when the carboxylate group on the side chain of the aspartic acid undergoes a nucleophilic attack on the carbonyl carbon of the adjacent peptide bond, leading not only to cleavage of the native peptide bond but also to the formation of an aspartic anhydride intermediate. The aspartic anhydride intermediate can then react with the N termini of other peptides at either carbonyl group of the anhydride. This leads to the formation of either a standard HIP, containing a classical peptide bond or an isomeric HIP in which the peptide is linked to the side chain of the aspartic acid through an isopeptide bond. Tautomerization of the anhydride through an enol intermediate could also occur, leading to the formation of corresponding D-enantiomers (D-HIP and D-isoHIP). However, since only two peaks for this HIP were observed in the NOD islets, we predicted that the primary HIPs present in these samples are the L-enantiomers (L-HIP and L-isoHIP). We hypothesized that the formation of such an anhydride intermediate is facilitated through the slightly acidic environment (pH 5.0–6.0) that is present within the lumen of insulin granules (11) and may therefore occur spontaneously. To determine if spontaneous HIP formation can occur, we conducted an *in vitro* experiment by coincubating HIP-precursor peptides (intact insulin C-peptide and the cleavage product of IAPP) at pH 5.0. We then analyzed samples by MS. Upon extracting the ion chromatogram for the expected HIP (*m/z* = 830.38), we observed two adjacent peaks, supporting the expectation that HIP-isomers had formed (Fig. 1*C*). The results show that coincubating the two peptides at pH 5.0 resulted in the formation of two products that have the same mass as the expected HIP and isoHIP.

**Table 1:** Aspartic acid HIPs found in NOD islets

Aspartic acid HIPs found in NOD islets	
First C-peptide aspartic acid	Second C-peptide aspartic acid
EVE**D**-TPVRSGTNPQ	EVEDPQVAQLELGGGPGAG**D**-GWR
EVE**D**-TPVRSGTNPQM	EVEDPQVAQLELGGGPGAG**D**-GWRPS
EVE**D**-NAARDPNRESLDF	EVEDPQVAQLELGGGPGAG**D**-LGAL
EVE**D**-NAARDPNRESLDFL	PQVAQLELGGGPGAG**D**-EVE
EVE**D**-EVAQQ	
EVE**D**-EVARQ	

List of HIPs formed at first and second aspartic acid (D) residues in C-peptide identified by Spectrum Mill analysis of NOD islets. D is in bold and highlights the aspartic acid residue at which the peptide crosslinking reaction occurs.

### Validating the *in vitro* formation of HIP isomers

**Figure 2:**
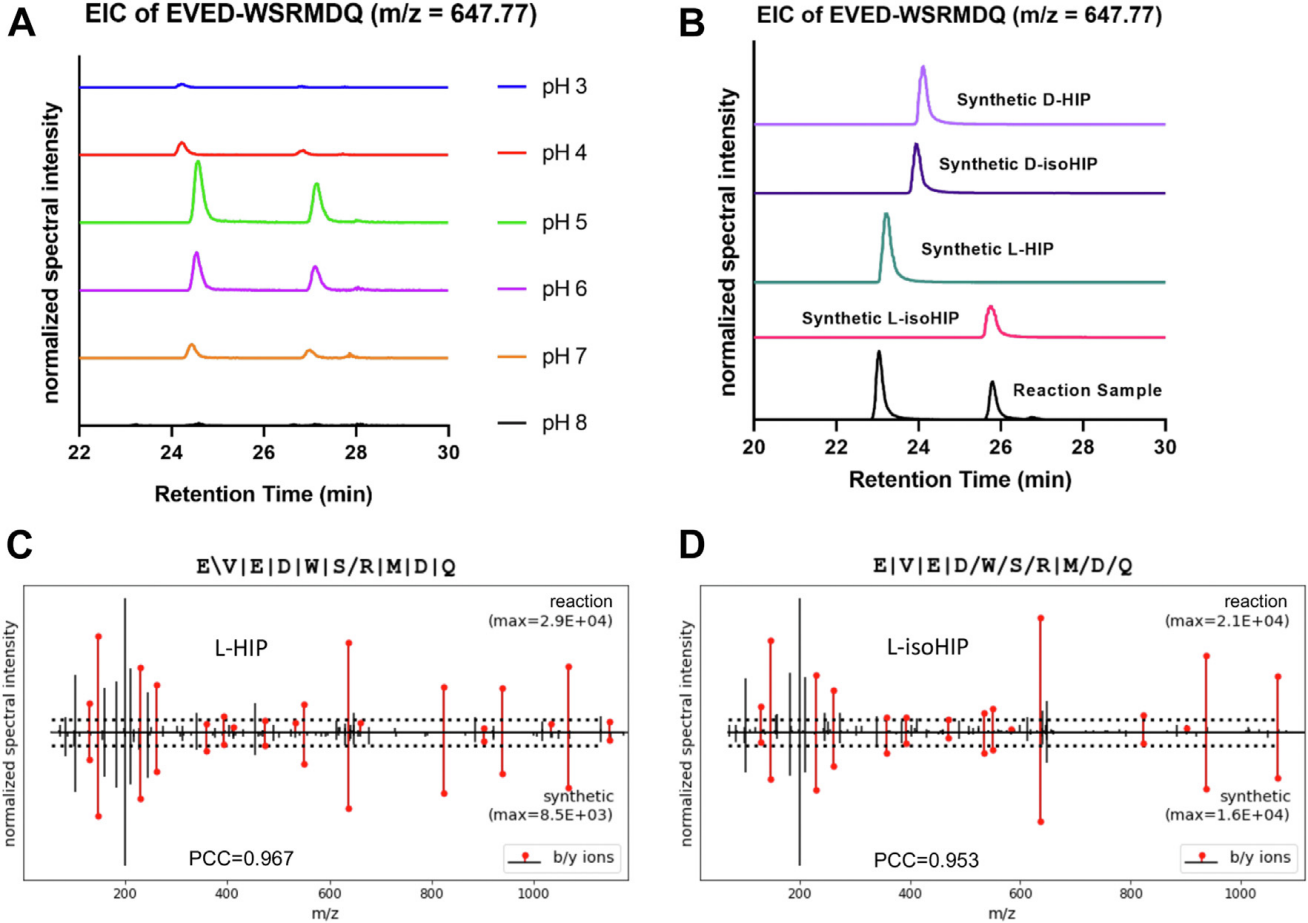
**Spontaneous *in vitro* murine (iso)HIP formation.***A*, EIC results for 24 h coincubation of full-length murine Ins2 C-peptide with ChgA fragment (WQ6) in the absence of any protease/enzyme over a pH range of 3 to 8. Peaks signify the presence of peptides with an *m/z* of 647.77 representing EVED-WSRMDQ. The normalized combined area under the curve (AUC) for each pH is as follows: pH 3, AUC 0.08; pH 4, AUC 0.32; pH 5, AUC 1; pH 6, AUC 0.58; pH 7, AUC 0.18; and pH 8, AUC 0.02. Experiment was done in triplicate. Aforementioned data show one representative experiment. *B*, EICs showing the elution profiles for the reaction HIPs and the synthetic validation HIPs: L-HIP, L-isoHIP, D-HIP, and D-isoHIP. *C* and *D*, the *in vitro* formation of (*C*) EVED-WSRMDQ HIP and (*D*) EVED-WSRMDQ isoHIP was validated using P-Vis protocol (12). The figure shows mirror plots generated during peptide validation comparing fragmentation spectra of reaction peptide to synthetic validation peptide. EIC, extracted ion chromatogram; HIP, hybrid insulin peptide.

To determine the impact of pH on the yield of this spontaneous reaction, we incubated murine C-peptide and a peptide fragment of ChgA (WQ6: WSRMDQ) over a broad pH range (pH 3.0–8.0). We chose this peptide as it was previously shown to participate in the formation of various HIPs that are detectable in murine islets (1, 5, 7). The results show that two HIP-isomers (sequence: EVED-WSRMDQ) had formed, as we detected two adjacent peaks for this peptide in the samples (Fig. 2*A*). The highest yield of HIPs, as measured by the total area under the curve of the EICs for HIP and isoHIP (*m/z* = 647.77), occurred at pH 5.0. We also observed HIP formation at pH 7.0, suggesting that this reaction may occur at physiological pH, albeit at lower yields. To determine which enantiomers (D or L) formed in the reaction samples, we commercially obtained and analyzed the four isomeric forms of the HIP (L-HIP, L-isoHIP, D-HIP, and D-isoHIP) using MS. As shown in Figure 1*B*, the L-HIP coeluted with the left peak in the reaction sample, whereas the L-isoHIP coeluted with the right peak. In line with our expectations, the synthetic forms of D-HIP and D-isoHIP did not coelute with the newly formed peptides in the reaction sample. Using the P-VIS validation protocol (12), we validated the identities of the L-HIP and L-isoHIP that formed in the reaction (Figs. S1 and S2). Figure 2*C* (L-HIP) and *D* (L-isoHIP) show mirror plots of the fragmentation spectra for the synthetic peptides and the peptides formed in the reaction samples. The correlation for the mirror plot spectra of the L-HIP and L-isoHIP achieved the threshold for validation with Pearson correlation coefficients (PCCs) of 0.967 (L-HIP) and 0.953 (L-isoHIP), respectively. It is noteworthy that the fragmentation spectra of the L-HIP and L-isoHIP only marginally differ from each other, highlighting the requirement of chromatographic coelution to validate the peptide identities. These results demonstrate that two types of HIPs, L-HIP and L-isoHIP, form spontaneously from precursor peptides in the absence of any protease or enzyme.

### Detecting (iso)HIPs in NOD islets

**Figure 3:**
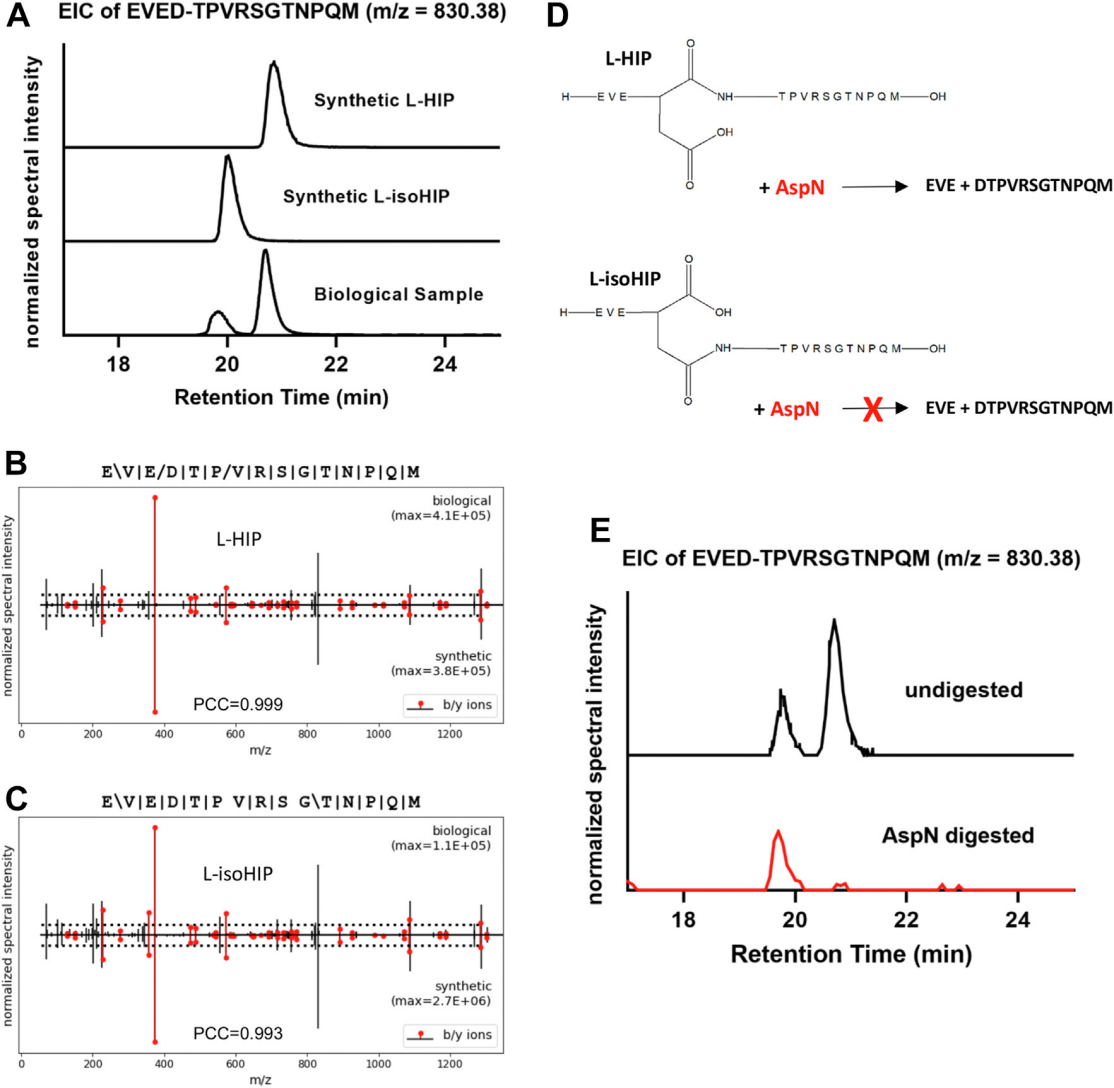
**Validation of (iso)HIP presence in NOD islets.***A*, EICs of EVED-TPVRSGTNPQM with an *m/z* of 830.38 showing the elution profiles for the biological HIPs and the synthetic validation HIPs: L-HIP and L-isoHIP. Experiment was done in triplicate. Aforementioned data show one representative experiment. *B* and *C*, the validation of (*B*) EVED-TPVRSGTNPQM HIP and (*C*) EVED-TPVRSGTNPQM isoHIP using P-Vis protocol (12). The figure shows mirror plots generated during peptide validation comparing fragmentation spectra of biological peptide to synthetic validation peptide. *D*, schematic showing digestion of EVED-TPVRSGTNPQM HIP *versus* EVED-TPVRSGTNPQM isoHIP by mass spectrometry protease AspN. *E*, EICs of EVED-TPVRSGTNPQM with an *m/z* of 830.38 showing result of (iso)HIP digestion by AspN. Peaks represent HIP/isoHIP presence in NOD islets prior to AspN digestion and after AspN digestion. L-HIP is digested by AspN, but L-isoHIP is not digested by AspN. Experiment was done in triplicate. Aforementioned data show one representative experiment. EIC, extracted ion chromatogram; HIP, hybrid insulin peptide; NOD, nonobese diabetic.

We next used the P-VIS protocol (12) to fully validate the identity of the L-HIP and L-isoHIP (sequence: EVED-TPVRSGTNPQM) that we detected in NOD islet samples (Figs. S3 and S4). Chromatographic elution profiles demonstrate that the synthetic L-HIP (Fig. 3*A*, *right peak*) and L-isoHIP (Fig. 3*A*, *left peak*) coeluted with the corresponding peaks detected in the biological samples (Fig. 3*A*). The correlation for the mirror plot spectra of the L-HIP and L-isoHIP achieved the threshold for validation as well with PCCs of 0.999 (L-HIP) and 0.993 (L-isoHIP), respectively (Fig. 3, *B* and *C*). To further substantiate the presence of isoHIPs in the biological sample, we exposed islet extracts to proteolytic digestion with the protease AspN, which cleaves peptide bonds that are N-terminally adjacent to D-residues with high specificity. However, this cleavage does not occur if the side chain of the aspartic acid is masked through an isopeptide bond (13), as is the case for isoHIPs. We therefore predicted that the AspN digest of peptides in the samples will only occur with the D-residue of the HIP but not the isoHIP (Fig. 3*D*). Following the digestion of our islet samples with AspN, we could only detect one EIC peak (Fig. 3*E*). As expected, the left peak remained intact, which is in line with the coelution profile for the isoHIP in Figure 3*A*. The results show that both HIP and isoHIP were present in NOD islets, substantiating the occurrence of the anhydride mechanism in beta-cells.

### Requirement of D-residues

Based on the mechanism shown in Figure 1*B*, we expect that D-residues are required for the mechanism to function. This is supported by the observation that several HIPs detected in NOD islets form at D-residues (Table 1). Aspartic acid and glutamic acid are very similar in structure, but glutamic acid has one extra methylene group in its side chain. Because of this additional methylene group, it is sterically unfavorable to form the cyclic anhydride intermediate. To test this hypothesis, we obtained an altered version of C-peptide in which the D-residue was replaced with a glutamic acid residue (D**→**E: EVE**E**PQVAQLELGGGPGAGDLQTLALEVAQQ). We incubated this modified form of C-peptide with the IAPP peptide fragment (TPVRSGTNPQM) at pH 5.0 and 37 °C for 24 h to determine if HIP/isoHIP formation would occur spontaneously (Fig. S5*C*). An EIC for the expected HIP/isoHIP (*m/z* = 837.39) sequence shows zero abundance (Fig. S5*D*), indicating that HIP formation did not occur with the modified peptide, whereas the HIP/isoHIP (*m/z* = 830.39) do form in the presence of wildtype C-peptide (Fig. S5*E*). These results confirm that D-residues are required for the spontaneous formation of HIPs/isoHIPs.

### Human HIP/isoHIP formation *in vitro* and HIP/isoHIP presence in human islets

**Figure 4:**
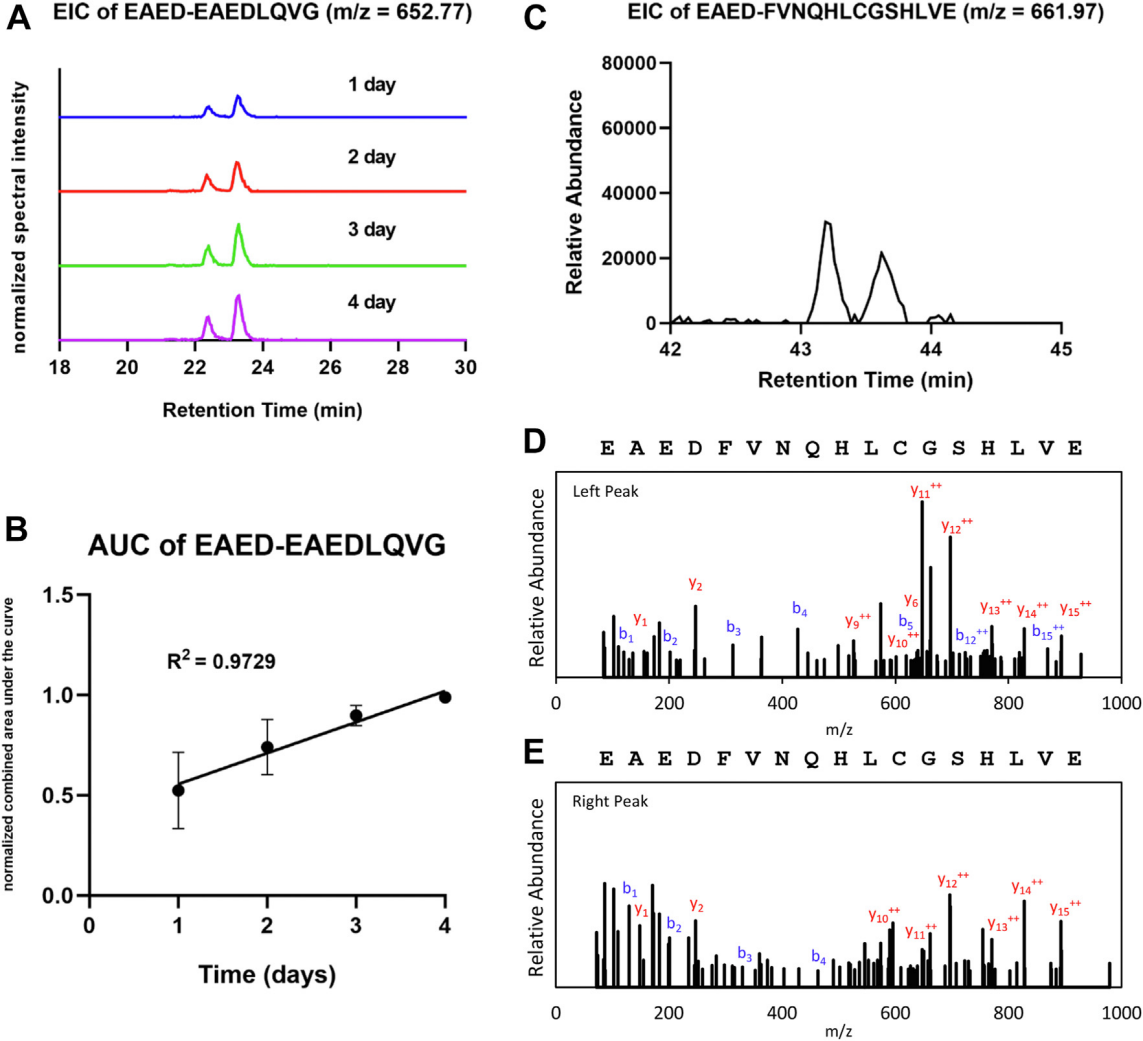
**Human (iso)HIP formation *in vitro* and the presence in human islets.***A*, EIC results for *in vitro* incubation of human C-peptide fragment EAEDLQVG in the absence of any protease/enzyme at pH 5.0 over the course of 4 days. Peaks signify relative abundance of peptides with an *m/z* of 652.77 representing EAED-EAEDLQVG. Experiment was done in triplicate. Aforementioned data show one representative experiment. *B*, fitted linear regression line for the yield of combined HIP/isoHIP formation overtime. Each dot represents the normalized combined area under the curve for HIP/isoHIP yield at each time point. Results shown are the mean ± SEM from three independent experiments. *C*, EIC of EAED-FVNQHLCGSHLVE with an *m/z* of 661.97 identifies two peaks representing HIP and isoHIP present in human islet samples. *D* and *E*, MS/MS fragmentation spectra of (*D*) *left peak* and (*E*) *right peak* of EAED-FVNQHLCGSHLVE showing the b (*blue*) and y (*red*) ions. EIC, extracted ion chromatogram; HIP, hybrid insulin peptide; MS, mass spectrometry.

Following the discovery of (iso)HIPs in NOD islet samples, we hypothesized that spontaneous isoHIP formation also occurs with human peptides. In order to experimentally verify this idea, we subjected a human C-peptide fragment that included a D-residue (EAEDLQVG) to incubation for several days at pH 5.0 and 37 °C. Using mass spectrometric analyses on these samples confirmed that HIP and isoHIP formation had occurred. The corresponding HIP sequence EAED-EAEDLQVG formed nonenzymatically from precursor peptide EAEDLQVG and was associated with two peaks indicating the formation of a standard HIP and isoHIP *in vitro*. We further hypothesized that the spontaneous formation of HIPs is an ongoing process and may lead to the accumulation of isoHIPs over time. To test this hypothesis, we incubated the precursor peptide for up to 4 days and removed daily aliquots for mass spectrometric analysis (Fig. 4*A*). As shown in Figure 4*B*, the yield of HIP/isoHIP increased linearly overtime. These results show that (iso)HIP formation is a continuous process, and the abundance of (iso)HIPs may thus serve as a marker of granular age. It also demonstrates that spontaneous (iso)HIP formation occurs with a human C-peptide fragment *in vitro*. Upon reviewing past mass spectrometric datasets of human islet samples, we successfully identified a novel (iso)HIP (EAED-FVNQHLCGSHLVE). This (iso)HIP consists of insulin C-peptide on the left and insulin B-chain on the right. The identification of this peptide in human islet samples required a specific experimental approach involving a partial GluC digest. This allowed us to detect the peptide sequence EAED-FVNQHLCGSHLVE through mass spectrometric analysis. As shown in Figure 4*C*, this (iso)HIP exhibits the distinct characteristic of having two proximal peaks in its elution profile, which is consistent with other (iso)HIPs identified. We then corroborated the identification of this same (iso)HIP in newly GluC-digested human islet samples. The (iso)HIP with the sequence EAED-FVNQHLCGSHLVE satisfies the rules for the identification of HIPs, which we previously established (see the Experimental procedures section) (5). The mass spectrometric fragmentation spectra for the two isomers are shown in Figure 4, *D* and *E*.

## Discussion

In this study, we present evidence for the spontaneous formation of peptide bonds in pancreatic beta-cells. This process involves the formation of a cyclic anhydride intermediate between the carboxylate group of an aspartic acid side chain and its C-terminally adjacent peptide bond, resulting in the cleavage of the peptide bond and the formation of an aspartic anhydride intermediate. The anhydride intermediate contains two carbonyl groups that can form a new bond upon the introduction of another peptide substrate, resulting in the formation of either a regular peptide bond or an isopeptide bond that links to the aspartic acid side chain. Through *in vitro* reactions, in which we coincubated HIP-precursor peptides, we established that the highest yield for this reaction occurs at pH 5.0, which is similar to the pH found in the soma of insulin granules (11). This finding suggests that insulin granules may be hotspots for the spontaneous formation of new peptide bonds. However, we also found that this new mechanism is not limited to acidic environments and can occur at neutral pH, although at lower yields.

We focused on C-peptide throughout this study because of mass spectrometric evidence indicating the occurrence of HIP formation specifically at the D-residues within C-peptide. Unlike insulin, which is tightly packed in the dense core of the insulin granule, C-peptide is more accessibly located in the surrounding granular halo (14) allowing it to interact with other peptides. Consequently, C-peptide has the potential to act as a prominent substrate for this spontaneous reaction, enabling the generation of detectable levels of (iso)HIPs.

To validate the presence of a HIP, which was previously detected by MS in murine islets (5, 10), and its corresponding isoHIP, we used chromatographic elution profiles of synthetic peptide standards to verify the identity of the isomers. This is a necessary step, because the fragmentation spectra of HIP and isoHIP are nearly identical and cannot be used to effectively differentiate between the two isomers. In addition, we subjected the biological peptides to proteolytic digestion using the metalloprotease AspN, which only cleaves the N-terminal peptide bond of D-residues if its side chain is not blocked, as it is in the case of isoHIPs. As expected, these data demonstrate that the biological isoHIP was not processed by AspN, confirming the presence of an isopeptide bond in murine islets. Furthermore, we discovered that the yield of (iso)HIPs increases linearly over time upon coincubation of precursor peptides, suggesting that isoHIPs may accumulate in older granules and serve as markers of granular age. While the role of granular age in the pathogenesis of T1D is currently unknown, the accumulation of (iso)HIPs in predisposed individuals could trigger an immune response upon reaching a hypothetical threshold.

Although various HIPs have been identified that contribute to the pathogenesis of T1D, the role of isoHIPs in this process has yet to be established. We predict that isoHIPs could become targets for autoreactivity within beta-cells by managing to avoid negative selection in the thymus. This evasion is expected to be due to the absence of necessary conditions within thymic epithelial cells, which play a vital role in the development and maturation of T cells. These conditions include high local concentrations of precursor peptides in a slightly acidic environment, both of which are required for the effective formation of (iso)HIPs through an anhydride mechanism. Thus, further research is necessary to investigate the role of these HIPs in the destruction of beta-cells. In addition, it is unclear whether spontaneous peptide bond formation occurs in other tissues, such as the myelin sheath, which is targeted for destruction in multiple sclerosis. Recent data indicate that significantly elevated levels of (nonhybrid) isoaspartic residues can be detected in myelin basic protein obtained from brain tissue of organ donors with multiple sclerosis (15). Unlike the formation of isoHIPs, the spontaneous formation of isoaspartic residues in myelin basic protein was proposed to involve a cyclic succinimide intermediate, which does not yield new peptide sequences. Conversely, in the case of (iso)HIP formation, an aspartic anhydride intermediate forms, allowing the formation of nongenomic peptide sequences. The formation of hybrid peptides in immune-prone tissues may reveal a mechanism of disease that is shared among autoimmune diseases. The mechanism described herein could allow for the generation of nongenomic peptides that are recognized as targets of the adaptive immune system in autoimmune diseases.

## Experimental procedures

### Murine islet isolation

Murine NOD islets were prepared from whole mouse pancreata at the University of Colorado Diabetes Research Center Tissue Procurement and Processing Core (K. Scott Beard). We observed no differences because of the sex of mice used for islet isolation. The animal study was reviewed and approved by University of Colorado Institutional Animal Care and Use Committee.

### Murine islet preparation for MS

Vials of 750 islet equivalents were resuspended in 50 μl of water and 50 μl of trifluoroethanol (TFE) and then sonicated for 5 min. The islets were then heated for 10 min at 95 °C and briefly vortexed every 2 min. The islets were sonicated for another 5 min, vortexed, then spun down at 17,000*g*. Supernatants from the islet homogenates were then run on an HPLC SEC column (Agilent—XBridge BEH200A 3.5 μm, 7.8 × 150 mm, PN186007639) with 25 mM ammonium acetate running buffer at 1 ml/min, and six fractions were collected in order to isolate peptides from proteins. Fractions 4 to 6 were then reduced and alkylated, and one set of fractions was AspN digested, whereas the second set of fractions was not digested prior to MS analysis. For the *in vivo* validation of (iso)HIPs, three separate islet pools were analyzed, each pool containing islets from four different mice. The experimental data plots are one representative of three separate experiments.

### Human islet preparation for MS

For human islet experiments, 1000 or 3000 islet equivalents, respectively, were thawed, resuspended in 50 μl of 1× PBS and 50 μl of TFE, and sonicated for 5 min. Islets were then heated at 95 °C for 10 min, vortexing every 2 min, and then sonicated for an additional 5 min. Samples were spun at 17,000*g* for 5 min to pellet debris. The supernatant of the 1000 islet sample was separated isocratically on a Waters 7.8 mm × 150 mm XBridge BEH SEC column (3.5 μm particles, 200 Å pores) with a 7.8 mm × 30 mm guard column using 25 mM ammonium acetate (pH ≈7) as the running buffer at a flow rate of 1 ml/min for 10 min. Four fractions of equal volume were collected between 3.8 and 7.4 min. The supernatant of the 3000 islet sample was separated by charge with ion-exchange chromatography using a GE HiTrap Q HP anion column (1 ml) with a 20 mM Tris running buffer. The material was eluted off the column over a 90 min period by a linear gradient of 20 mM Tris–1 M NaCl at a flow rate of 0.25 ml/min to collect 16 fractions. Fractions were then dried under vacuum at 55 °C. Dried fractions were reconstituted with 100 mM ammonium bicarbonate (pH 8.0) and TFE. To reduce and alkylate the fractions, 20 mM DTT was added to each fraction and then incubated at 60 °C for 45 min. Subsequently, 20 mM iodoacetamide was added to each fraction and incubated at room temperature in the dark for 60 min. Alkylation was then stopped by another addition of 20 mM DTT and incubated for 60 min at room temperature in the dark. The fractions were then diluted to 25 mM ammonium bicarbonate for digestion. Human islet samples were digested with GluC (Thermo) at a final enzyme:protein ratio of 1:20 (w/w), and reactions were incubated for 4 h at 37 °C. After digestion, the 16 ion-exchange chromatography fractions were first desalted by Pierce C-18 Spin columns (Thermo Scientific), and then all fractions were dried under a vacuum at 55 °C. Samples were reconstituted in loading buffer (2.7% acetonitrile/0.1% formic acid/water), sonicated, and spun at 17,000*g* to remove any insoluble material. The supernatant was then analyzed by LC–MS/MS.

### *In vitro* (iso)HIP reactions

The reactions for *in vitro* HIP and isoHIP formation were incubated at 37 °C for the specified time and using McIlvaine buffers containing different concentrations of sodium phosphate and citric acid for the different pHs specified. *In vitro* mouse reactions included 0.5 mM WQ6, a fragment of the naturally occurring ChgA cleavage product, WE14, and 0.5 mM insulin 2 C-peptide. *In vitro* human reactions included insulin C-peptide fragment EAEDLQVG at 0.7 mM.

### AspN digestion reactions

Homogenized murine islets were incubated overnight at 37 °C in the presence or in the absence of the protease AspN. AspN is a widely used enzyme/endoprotease for the specific cleavage of peptides at the N terminus of D-residues for MS analysis. Synthetic EVEDTPVRSGTNPQM HIP and isoHIP were also incubated in the presence of AspN for 24 h at 37 °C (Fig. S5, *A* and *B*). Samples were dried down in a SpeedVac at 55 °C and reconstituted with 3% acetonitrile + 0.1% formic acid for MS analysis.

### MS

NOD islets were prepared for LC–MS/MS analyses as described elsewhere (5). *In vitro* reactions and prepared NOD islets were analyzed by LC–MS/MS using an Agilent 1200 series UHPLC system with a nanoflow adapter and an Agilent 6550 Q-TOF equipped with a nano-ESI source. Online separation was accomplished by reversed-phase liquid chromatography using a Thermo Acclaim PepMap 100 C18 trap column (75 μm × 2 cm; 3 μm particles; 100 Å pores) and Thermo Acclaim PepMap RSLC C18 analytical column (75 μm inner diameter; 2 μm particles; 100 Å pores) in a trap forward-elute configuration using a water/acetonitrile gradient (buffer A: 0.1% formic acid in water; buffer B: 0.1% formic acid and 90% acetonitrile in water). A detailed description of the MS data collection was previously published (12).

### HIP database analysis

Data were analyzed using the Spectrum Mill software with the SwissProt mouse/human database as well as HIP-specific databases that were generated using an in-house computer algorithm. The mouse HIP database contained each possible C-terminal truncation of insulin C-peptide linked to predicted naturally occurring cleavage products of insulin (1&2), ChgA, IAPP, secretogranin 1, and neuropeptide Y, making a total of 3000 peptides. More detail of this database and our established set of confidence criteria is provided elsewhere (5). The human HIP database contained all 30 HIP sequences that can form between C-peptide fragments on the left side linked to intact C-peptide on the right side. Search settings were as follows: instrument = Agilent ESI Q-TOF; precursor mass tolerance = ±10 ppm; product ion mass tolerance = ±20 ppm; and digest = no enzyme. Matches were considered valid if the following thresholds were satisfied: score >10, percentage scored peak intensity >70%, and rank one minus rank 2 (R1–R2) score >2.5.

### Confidence criteria for putative HIP identifications (5)

(1) Spectrum cannot be confidently matched to any peptides in a traditional protein database. (2) Spectrum matches with high confidence and little ambiguity to a peptide in the HIP database. (3) Peptide match corresponds to the proteolytic digest performed in sample preparation. (4) The left peptide and right peptide regions each contain at least two amino acid residues. (5) The spectrum contains sufficient b-/y-ion coverage of both the left and right peptide regions of the HIP.

### HIP/isoHIP validation

The synthetic versions of the HIP/isoHIP sequences found in our samples were obtained for validation purposes. These validation peptides and the biological/reaction samples were each spiked with a panel of internal standard peptides (ISPs). The biological/reaction samples were run sequentially with one peak targeted at a time to isolate spectra for each (iso)HIP for validation. The in-house developed software program, PSM validator, was then used to compare the fragmentation spectrum of the biological/reaction peptide to the spectrum of the validation peptide by calculating the PCC, which was also calculated for each of the ISPs. The PCCs of the ISPs were then used to calculate a 95% confidence interval. If the PCC of the peptide of interest falls within the 95% confidence interval, then it is considered a fully validated peptide. A detailed description of the P-VIS validation approach with the PSM validator algorithm was previously published (12).

### Synthetic peptides and ISPs

PROCAL (JPT Peptide Technologies) was used as ISPs. All other synthetic peptides were obtained from Genescript or SynPeptide at chromatographic purities of 95% or above.

## Data availability

The MS proteomics data have been deposited at the ProteomeXchange Consortium *via* the Proteomics Identifications (PRIDE) partner repository with the dataset identifier PXD041616. The PSM_validator software analyzed during the current study is available freely at https://github.com/Delong-Lab/PSM_validator/releases under the Creative Commons Attribution 4.0 International Public License.

PRIDE Reviewer account details: Username: reviewer_pxd041616@ebi.ac.uk and Password: mopOcxcE.

## Supporting information

This article contains supporting information (12).

## Conflict of interest

T. D. is the guarantor of this work and, as such, had full access to all the data in the study and takes responsibility for the integrity of the data and the accuracy of the data analysis. The authors declare that they have no conflicts of interest with the contents of this article.
