# Supplementary material for: Hybrid insulin peptide isomers spontaneously form in pancreatic beta-cells from an aspartic anhydride intermediate
Source: J Biol Chem. 2023 Sep 19;299(11):105264. doi: 10.1016/j.jbc.2023.105264 (PMC10590738; doi:10.1016/j.jbc.2023.105264)
Supplement: Supporting information [file mmc1.docx]

**Manuscript title:** Hybrid insulin peptide isomers spontaneously form in pancreatic beta-cells from an aspartic anhydride intermediate

**Authors:** Samantha Crawford, Mylinh Dang, Cole Michel, Jason Groegler, Roger L. Powell, Anita C. Hohenstein, Kaitlin Reyes, Kathryn Haskins, Timothy A. Wiles, and Thomas Delong

**Supporting Information Material:**

**Figure S1.** In vitro L-HIP formation validation

**Figure S2.** In vitro L-isoHIP formation validation

**Figure S3.** NOD islet L-HIP validation

**Figure S4.** NOD islet L-isoHIP validation

**Figure S5.** HIP/isoHIP control reactions

**
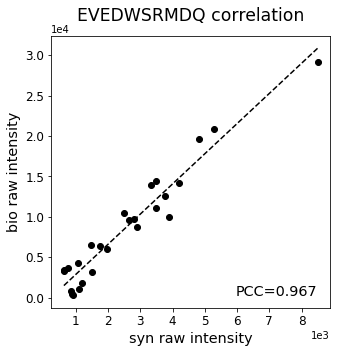
**

**A**

**B**

**C**

**D**

**E**

**F**

**
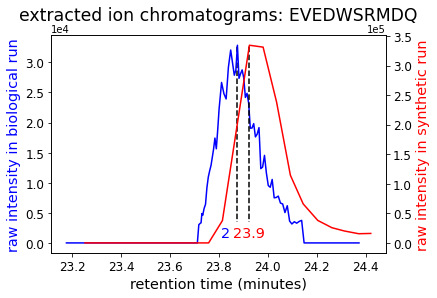
**

reaction

rxn

**
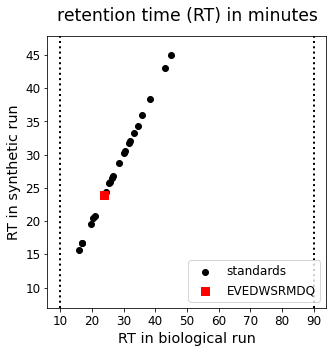

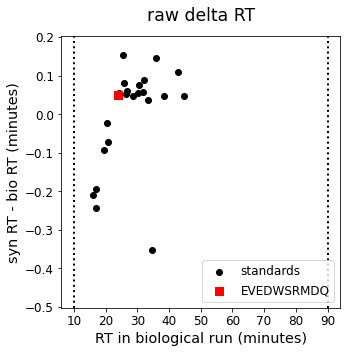
**

rxn

reaction

reaction

**
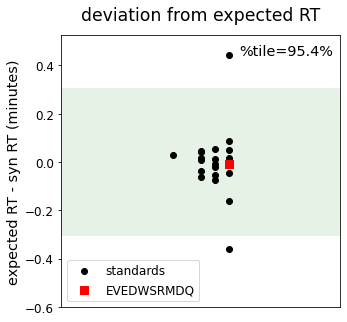

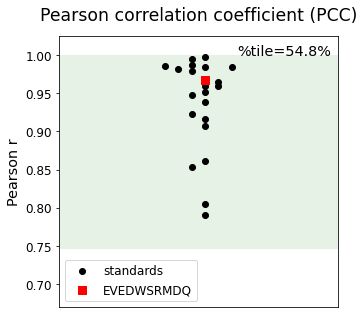
**

**Figure S1. P-VIS^12^ validation plots of spontaneous *in vitro* EVEDWSRMDQ L-HIP formation.** A) Raw intensities in the reaction spectrum and the validation spectrum for all fragmentation peaks of EVEDWSRMDQ included in calculation of the Pearson correlation coefficient (PCC). B) Extracted ion chromatogram showing the raw retention time for the reaction peptide (23.85 min) and the validation peptide (23.9 min). C) Difference in RT for each of the internal standard peptides (ISPs) between the two sample runs (reaction vs synthetic) and the RT difference between the reaction peptide and the validation peptide. Vertical dotted lines indicate the time range considered in RT analysis (10–90 min). D) RT of the ISPs and EVEDWSRMDQ L-HIP in the synthetic sample plotted against the RT of the ISPs and EVEDWSRMDQ L-HIP in the reaction sample. Vertical dotted lines indicate the time range considered in RT analysis (10–90 min). E) Linear spline model based on the ISP data was used to model the relationship between RT in the two sample runs. The model was used to predict the RT of each peptide in the validation sample run based on the RT in the reaction sample run. Green shading indicates the 95% prediction interval based on two-tailed analysis. The difference between the reaction peptide and the validation peptide is also shown, and the percentile (%tile) is reported. F) Distribution of PCCs for ISPs. Green shading indicates the 95% prediction interval based on one-tailed analysis. The PCC comparing the reaction spectrum and the validation spectrum is also shown, and the percentile (%tile) is reported.

**
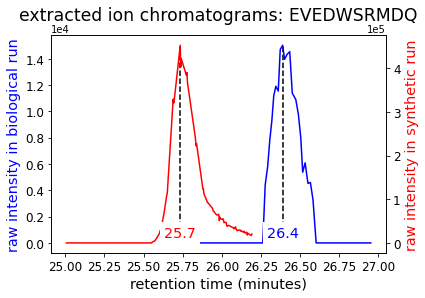

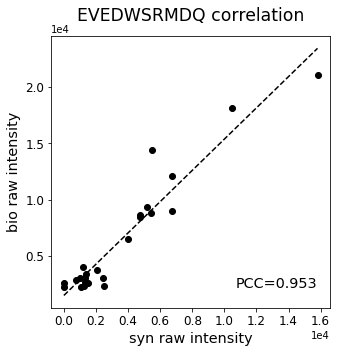
**

**A**

**B**

**C**

**D**

**E**

**F**

reaction

rxn

**
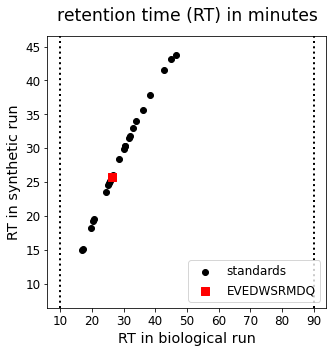

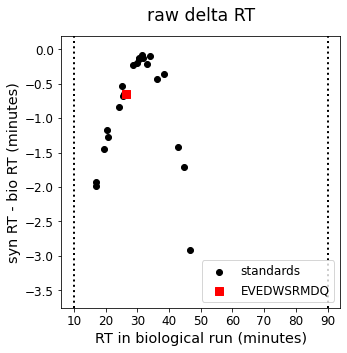
**

rxn

reaction

reaction

**
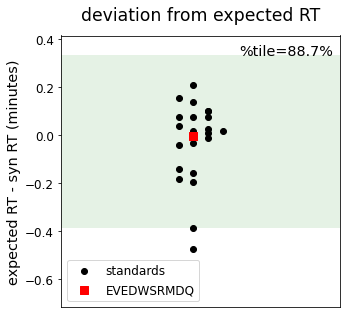

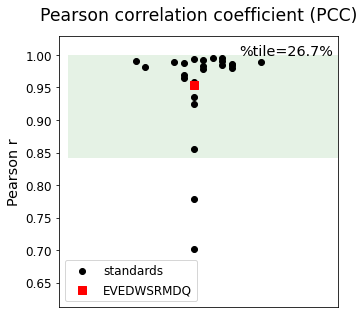
**

**Figure S2. P-VIS^12^ validation plots of spontaneous *in vitro* EVEDWSRMDQ L-isoHIP formation.** A) Raw intensities in the reaction spectrum and the validation spectrum for all fragmentation peaks of EVEDWSRMDQ included in calculation of the Pearson correlation coefficient (PCC). B) Extracted ion chromatogram showing the raw retention time for the reaction peptide and the validation peptide. C) Difference in RT for each of the internal standard peptides (ISPs) between the two sample runs (reaction vs synthetic) and the RT difference between the reaction peptide and the validation peptide. Vertical dotted lines indicate the time range considered in RT analysis (10–90 min). D) RT of the ISPs and EVEDWSRMDQ L-isoHIP in the synthetic sample plotted against the RT of the ISPs and EVEDWSRMDQ L-isoHIP in the reaction sample. Vertical dotted lines indicate the time range considered in RT analysis (10–90 min). E) Linear spline model based on the ISP data was used to model the relationship between RT in the two sample runs. The model was used to predict the RT of each peptide in the validation sample run based on the RT in the reaction sample run. Green shading indicates the 95% prediction interval based on two-tailed analysis. The difference between the reaction peptide and the validation peptide is also shown, and the percentile (%tile) is reported. F) Distribution of PCCs for ISPs. Green shading indicates the 95% prediction interval based on one-tailed analysis. The PCC comparing the reaction spectrum and the validation spectrum is also shown, and the percentile (%tile) is reported.

**
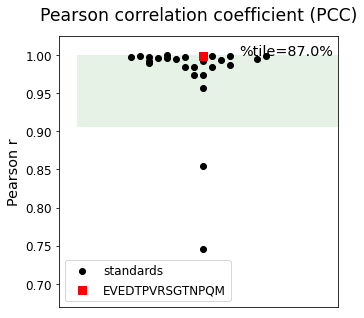

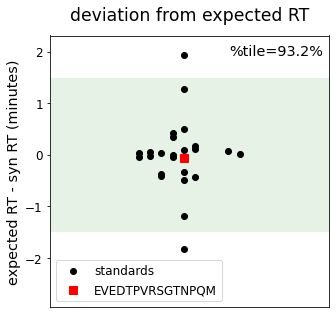

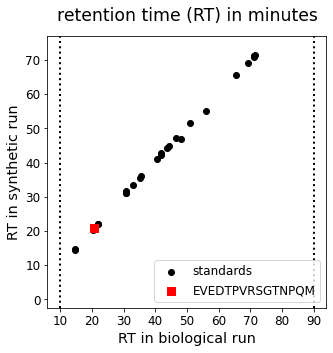

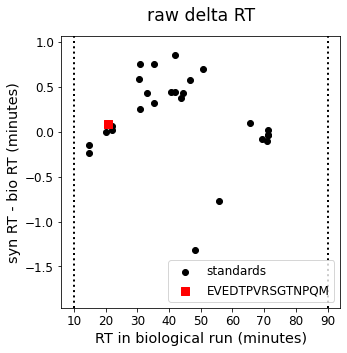

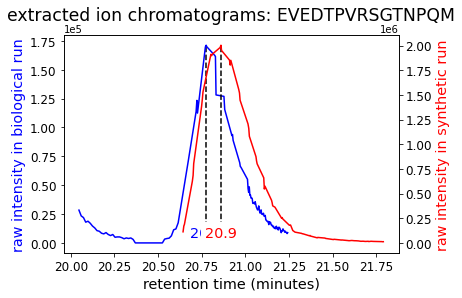

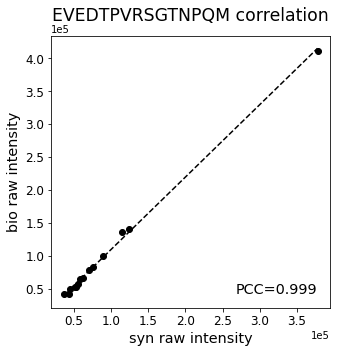
**

**A**

**B**

**C**

**D**

**E**

**F**

**Figure S3. P-VIS^12^ validation plots of EVEDTPVRSGTNPQM L-HIP in NOD islets.** A) Raw intensities in the biological spectrum and the validation spectrum for all fragmentation peaks of EVEDTRPVRSGTNPQM L-HIP included in calculation of the Pearson correlation coefficient (PCC). B) Extracted ion chromatogram showing the raw retention time for the biological peptide (20.8 min) and the validation peptide (20.9 min). C) Difference in RT for each of the internal standard peptides (ISPs) between the two sample runs (biological vs synthetic) and the RT difference between the biological peptide and the validation peptide. Vertical dotted lines indicate the time range considered in RT analysis (10–90 min). D) RT of the ISPs and EVEDTPVRSGTNPQM L-HIP in the synthetic sample plotted against the RT of the ISPs and EVEDTPVRSGTNPQM L-HIP in the biological sample. Vertical dotted lines indicate the time range considered in RT analysis (10–90 min). E) Linear spline model based on the ISP data was used to model the relationship between RT in the two sample runs. The model was used to predict the RT of each peptide in the validation sample run based on the RT in the biological sample run. Green shading indicates the 95% prediction interval based on two-tailed analysis. The difference between the biological peptide and the validation peptide is also shown, and the percentile (%tile) is reported. F) Distribution of PCCs for ISPs. Green shading indicates the 95% prediction interval based on one-tailed analysis. The PCC comparing the biological spectrum and the validation spectrum is also shown, and the percentile (%tile) is reported.

**
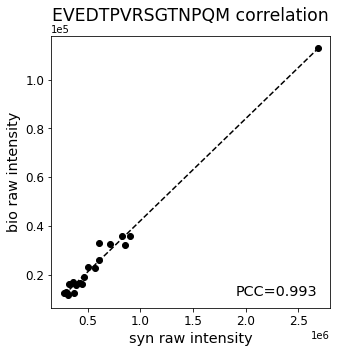
**

**A**

**B**

**C**

**D**

**E**

**F**

**
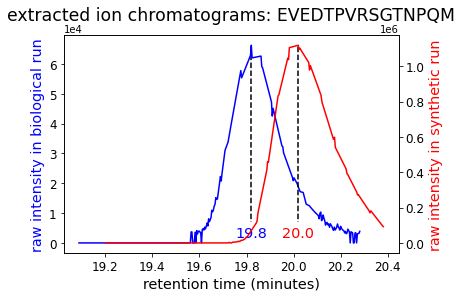
**

**
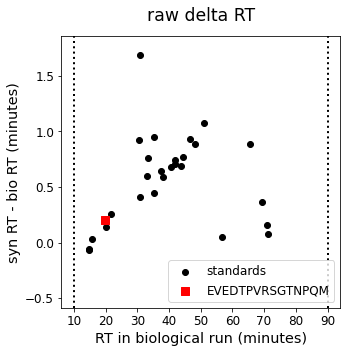

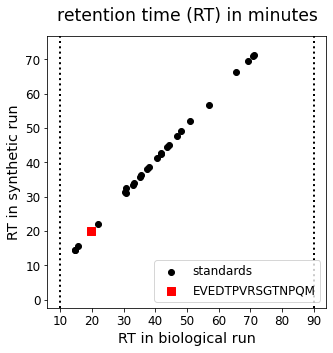

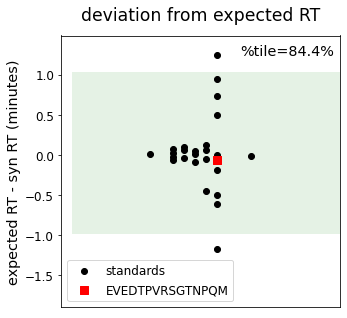

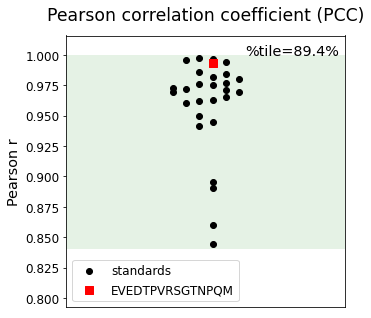
**

**Figure S4. P-VIS^12^ validation plots of EVEDTPVRSGTNPQM L-isoHIP in NOD islets.** A) Raw intensities in the biological spectrum and the validation spectrum for all fragmentation peaks of EVEDTRPVRSGTNPQM L-isoHIP included in calculation of the Pearson correlation coefficient (PCC). B) Extracted ion chromatogram showing the raw retention time for the biological peptide and the validation peptide. C) Difference in RT for each of the internal standard peptides (ISPs) between the two sample runs (biological vs synthetic) and the RT difference between the biological peptide and the validation peptide. Vertical dotted lines indicate the time range considered in RT analysis (10–90 min). D) RT of the ISPs and EVEDTPVRSGTNPQM L-isoHIP in the synthetic sample plotted against the RT of the ISPs and EVEDTPVRSGTNPQM L-isoHIP in the biological sample. Vertical dotted lines indicate the time range considered in RT analysis (10–90 min). E) Linear spline model based on the ISP data was used to model the relationship between RT in the two sample runs. The model was used to predict the RT of each peptide in the validation sample run based on the RT in the biological sample run. Green shading indicates the 95% prediction interval based on two-tailed analysis. The difference between the biological peptide and the validation peptide is also shown, and the percentile (%tile) is reported. F) Distribution of PCCs for ISPs. Green shading indicates the 95% prediction interval based on one-tailed analysis. The PCC comparing the biological spectrum and the validation spectrum is also shown, and the percentile (%tile) is reported.

**B**

**A**

**(IAPP1)**

**C**

**EVEEPQVAQLELGGGPGAGDLQTLALEVAQQ + TPVRSGTNPQM**

**(C-Peptide)**

**X**

**pH5, 24hr**

**EVEETPVRSGTNPQM**

**(HIP + isoHIP)**

**D**

**E**

*zero abundance*

**Figure S5. HIP/isoHIP control reactions.** A) Extracted ion chromatograms (EICs) of synthetic EVED-TPVRSGTNPQM L-isoHIP with m/z of 830.38 showing result of L-isoHIP digestion by AspN. Peaks represent EVED-TPVRSGTNPQM L-isoHIP presence in reaction sample prior to AspN digestion and after AspN digestion. Synthetic L-isoHIP is not digested by AspN, just as L-isoHIP was not digested by AspN in the biological NOD islet sample (see Figure 3e). B) EICs of synthetic EVED-TPVRSGTNPQM L-HIP with m/z of 830.38 showing result of L-HIP digestion by AspN. Peaks represent EVED-TPVRSGTNPQM L-HIP presence in reaction sample prior to AspN digestion and after AspN digestion. Synthetic L-HIP is digested by AspN, just as L-HIP was digested by AspN in the biological NOD islet sample (see Figure 3e). C) Schematic showing the experimental set up of D🡪E mutated C-peptide co-incubated with IAPP fragment (IAPP1) in vitro with the absence of any protease/enzyme at pH 5.0 for 24hr. D) EIC of EVEE-TPVRSGTNPQM with m/z of 837.39 showing zero abundance of the theoretical product peptide from the experimental set up of Supp. Figure 5C. E) EIC of EVED-TPVRSGTNPQM with m/z of 830.38 showing two peaks correlating to the formation of HIP and isoHIP from the experimental set up of full length murine Ins2 C-peptide co-incubated with IAPP fragment (IAPP1) *in vitro* with the absence of any protease/enzyme at pH 5.0 for 24hr (positive control reaction for Supp. Figure 5C/D). All control reactions were done in triplicate. Above data shows one representative experiment.
